# Lessons from a ten-year-long journey: building a student-driven computational biology society across Turkey

**DOI:** 10.12688/f1000research.107886.1

**Published:** 2022-01-26

**Authors:** Yasin Kaya, Tülay Karakulak, Cemil Can Saylan, E. Ravza Gür, Engin Tatlıdil, Sevilay Güleşen, Fatma Betül Dinçaslan, Handan Melike Dönertaş

**Affiliations:** 1 Department of Biology, Institute of Science, Hacettepe University, Beytepe, Ankara, 06800, Turkey; 2 Department of Molecular Life Sciences, University of Zurich, Zurich, Switzerland; 3 Department of Pathology and Molecular Pathology, University Hospital Zurich, Zurich, Switzerland; 4 Computational Science and Engineering Department, Informatics Institute, Istanbul Technical University, Sariyer, Istanbul, 34467, Turkey; 5 MRC WIMM Centre for Computational Biology, MRC Weatherall Institute of Molecular Medicine, Radcliffe Department of Medicine, University of Oxford, Oxford, OX3 9DS, UK; 6 MRC Molecular Haematology Unit, MRC Weatherall Institute of Molecular Medicine, Radcliffe Department of Medicine, University of Oxford, Oxford, OX3 9DS, UK; 7 Department of Molecular Biology and Genetics, Izmir Institute of Technology, Urla, Izmir, 35430, Turkey; 8 Core Program, Kadir Has University, Fatih, Istanbul, Turkey; 9 Department of Biomedical Engineering, National University of Singapore, Singapore, 117583, Singapore; 10 Institute for Health Innovation and Technology, Singapore, 117599, Singapore; 11 Leibniz Institute on Aging, Fritz Lipmann Institute (FLI), Jena, 07745, Germany

**Keywords:** student council, iscb, turkey, 10 years

## Abstract

The Regional Student Group Turkey (RSG-Turkey) is officially associated with the International Society for Computational Biology (ISCB) Student Council (SC). At the RSG-Turkey, we aim to contribute to the early-career researchers in computational biology and bioinformatics fields by providing opportunities for improving their academic and technical skills in the field. Over the last ten years, we have built a well-known student-driven academic society in Turkey that organizes numerous events every year and continues to grow with over 650 current members. Celebrating the 10th anniversary of RSG-Turkey, in this communication, we share our experiences, five main lessons we learned, and the steps to establish a long-standing academic community: having a clear mission, building a robust structure, effective communication, turning challenges into opportunities, and building collaborations. We believe that our experiences can help students and academics establish long-standing communities in fast-developing areas like bioinformatics.

## Introduction

The Regional Student Group Turkey (RSG-Turkey) is a member of the International Society for Computational Biology (ISCB) Student Council (SC) and is a non-profit community consisting of early-career researchers interested in computational biology. ISCB-SC is a global community that assists with the development of computational biologists by promoting and offering scientific events, networking opportunities, training, and educational resources. ^1^^–^^3^ RSGs were initiated to promote computational biology and events in local geographical regions. ^4^ Today, there are more than 30 RSGs worldwide and they not only organize local events but interact and are part of a large network. ^5^ Following the principle that “unity makes strength”, *i.e.,* greater things can be achieved by teamwork and collaboration, RSG-Turkey was seeded in December 2011 to bring young computational biologists together. Our community aims to provide a framework to share the knowledge and experience across institutions and promote collaborations within and beyond Turkey towards open science goals.

**Figure 1.**
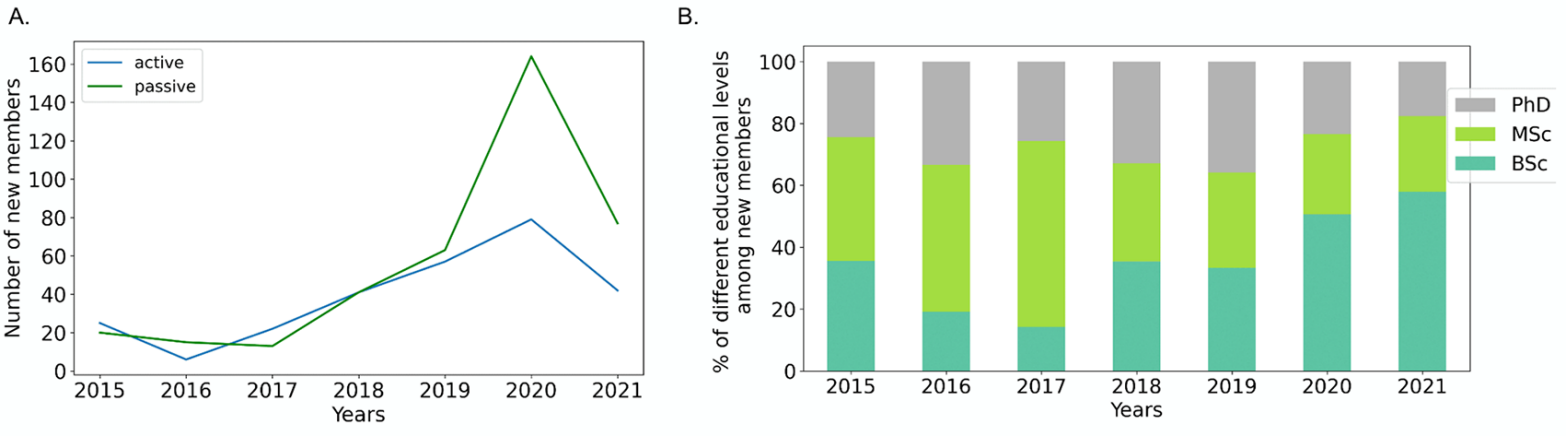
(A) The number of new active and passive members across years. (B) Percentage of educational levels among new RSG-Turkey members across years. Numbers in the current year (2021) are incomplete.

Computational biology and bioinformatics are fast-developing research areas in Turkey. In the past, the field of bioinformatics was only included in the undergraduate curriculum as a definition. Advanced technology has led to easier data generation and a plethora of publicly available data. However, theoretical work, re-analysis of publicly available data, and bioinformatics gains even a more important position in countries like Turkey, where the rate of data generation is not comparable to trends in the highly developed world. The need for experts in this area in Turkey has led to the development of new graduate programs on bioinformatics at more than ten universities. The rapid development also enabled industrial growth with 109 companies specializing in bioinformatics. ^6^ Despite an increase in the number of postgraduate schools, comprehensive undergraduate education on bioinformatics is still limited, with only one program across the whole country. Not having the required multi-disciplinary background limits students from immediate engagement with research in graduate school and sometimes even limits their interest to pursue the relevant graduate degrees as they are often required to take additional courses during a “scientific preparation year”. As RSG-Turkey, we aim to bridge this gap by bringing students interested in bioinformatics and computational biology together as early as possible, providing them with an environment to learn from each other and engage with the academic society. Our efforts have started to bear fruit and we have started to attract not only more members ( Figure 1A) but more bachelor (BSc) students since 2019 ( Figure 1B).

**Figure 2.**
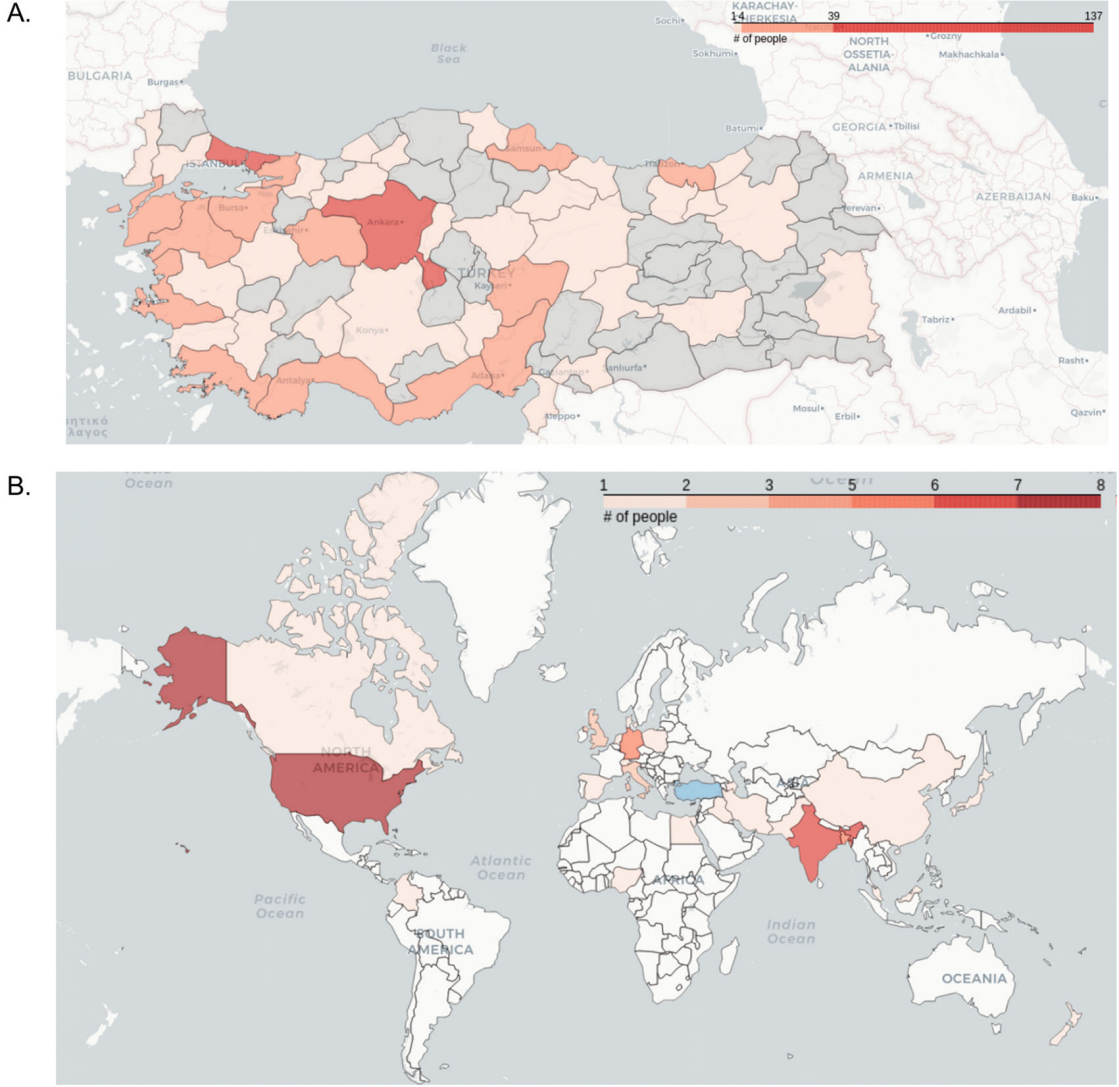
RSG-Turkey members in (A) Turkey and (B) worldwide.

Events organized by RSG-Turkey are the result of collective work among members. We reunite people from different career levels, undergrad, master, PhD and young principal investigators. These early-career researchers in computational biology have been involved with academic activities such as scientific meetings and conferences and actively participated in regional or international student societies for many years, leading to building an academic network around students at universities to guide them on their careers. Our members are located across all regions of Turkey and the number of members reflects the number of universities and active bioinformatics researchers in each city ( Figure 2A). Furthermore, our members are not restricted to researchers residing in Turkey but involve many scientists who are connected to scientific society in Turkey ( *e.g.,* they completed their undergraduate degree in Turkey but are now abroad or researchers residing in neighbouring countries without RSGs). Indeed, our members are distributed across the globe ( Figure 2B), bringing diverse experiences across different scientific environments together. Importantly, RSG-Turkey also provides a framework for ECRs living abroad to continue interacting with and contributing to the scientific environment in the country. Five of our ten workshops were indeed given by our members who study or work abroad.

**Figure 3.**
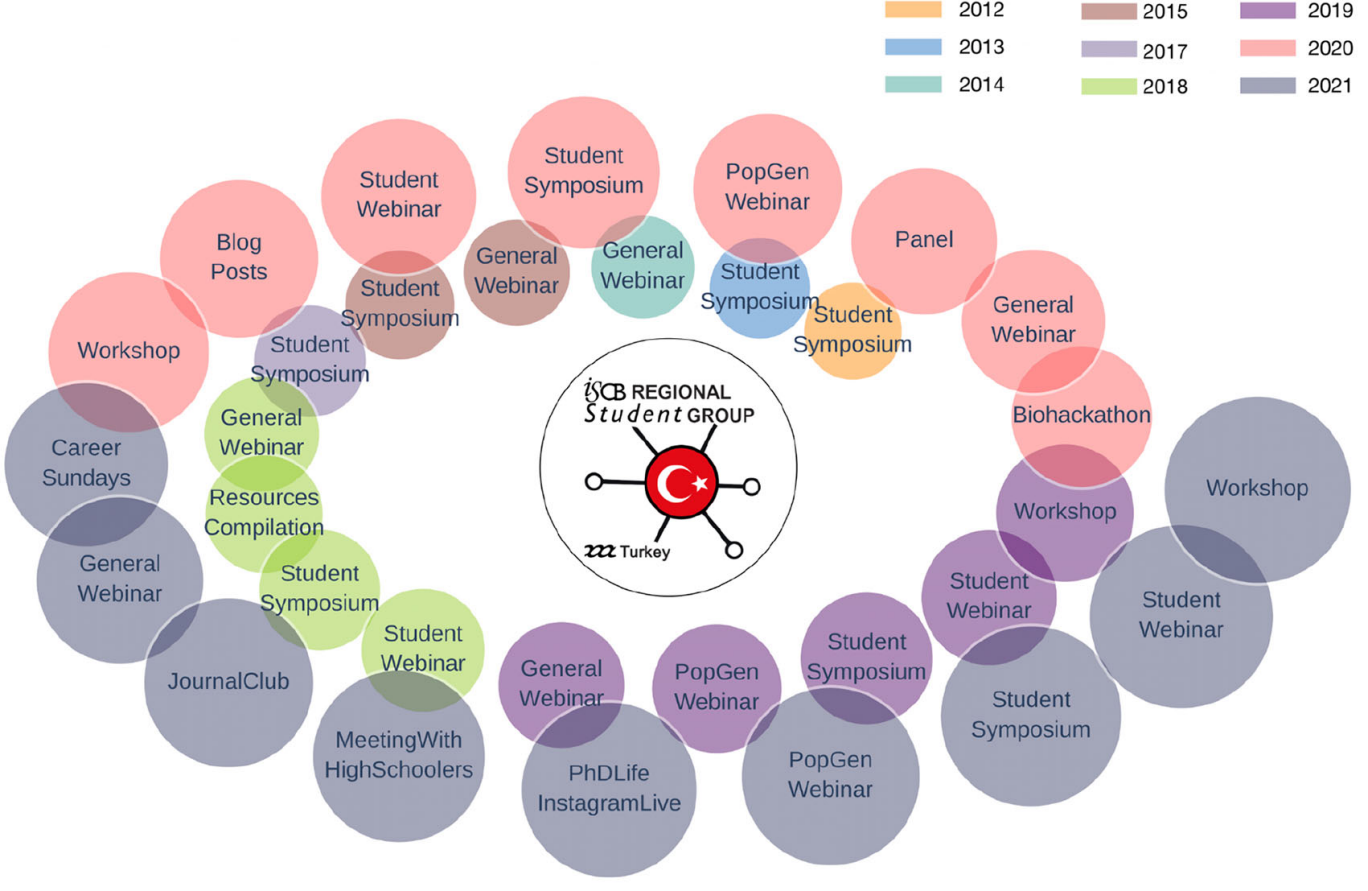
Main events and activities of RSG-Turkey across years.

RSG-Turkey has become one of the most well-known academic societies in Turkey. As RSG-Turkey, we offer ECRs various opportunities to come together, interact, and learn. Historically, our main activities have been the annual student symposium and free webinars. Webinars and student symposiums organized in the first five years of RSG-Turkey and the fast development of computational biology have attracted new students to our society. Our activities have expanded during the last few years with the addition of workshops, a biohackathon, panels, tutorials and short articles published on our website, career events, journal clubs, and outreach activities with high school students ( Figure 3). Moreover, brainstorming following short discussions and having more members paved the way for compiling resources for computational biology and bioinformatics to contribute to open science. To provide guidance on career and life during and after graduate school, we launched an Instagram series on “PhD Life” and YouTube series for “Career Sessions”, where we hosted experienced researchers in academia and industry. Thus, RSG-Turkey has provided excellent opportunities for students to expand their academic network, create, improve, share their knowledge on different bioinformatics topics, innovate, and train younger generations.

RSG-Turkey celebrated its 10 ^th^ year with more than 650 members this year. In this paper, we share five lessons we have learned from successfully maintaining and advancing a student-driven academic society over a long period.

## Lesson 1: Clearly define the mission and values for the society but also allow adaptation to change in the long-term

Member turnover in a student-driven society is relatively high. Agreeing on a mission and well-defined values ensure long-term maintenance of the culture within the community. Historically, our members have mainly consisted of Master's students who took active roles during the early stages of their PhDs, *i.e.,* only two to three years each. Without clearly defined goals, it is easy for the community to drift away. In recent years, we succeeded in attracting students from undergrad programs who continue to be a part of society even after completing their PhDs. However, the challenge still exists. At RSG-Turkey, our primary mission is to establish a collaborative environment in and around Turkey that fosters sharing knowledge among early-career researchers working in the area of computational biology. While doing so, a few values have emerged over the years. We now make sure these are followed as moral pillars of our community: democratizing knowledge and access to opportunities, fostering diversity, and collaboration. These principles provide the overall framework while allowing new members to explore their ideas and adapt to emerging trends and needs. For example, we provide a platform for our members to freely share their knowledge and experience in various formats (e.g., blog posts, webinars, online discussions) based on the community’s needs. In other words, RSG-Turkey is a place where we encourage our members to share their expertise in their own way and schedule. Luckily, this flexibility resulted in numerous posts and tutorials published on our website, ^7^ written by the members who are active researchers in the areas they write about. Similarly, although our initial focus was introducing computational biology to students and creating training opportunities, career-focused events have recently become necessary with new universities providing bioinformatics degrees. We thus started free career events streamed on YouTube ( https://www.youtube.com/c/RSGTurkey) and Instagram ( https://www.instagram.com/rsgturkey/) to introduce alternative career paths and provide example career trajectories to the participants. Therefore, allowing the evolution within the group in a systematic way enabled more students to engage, contribute and be part of the big data revolution in biology and computer-related disciplines.

## Lesson 2: Build a fluid, robust and bottom-up structure

No matter how good the ultimate goal of a community is, it is shaped by the profile of its members. In RSG-Turkey, we believe the society would only thrive if the members are diverse but align well on the fundamental values and the society's mission. Overall, our only criterion is being an active researcher or a strong interest in bioinformatics. However, we have introduced two types of membership: i) passive members, who attend events and are included in the announcements, and ii) active members, who organize the events. We require a motivation letter from active members to get to know them and see how well we can align in terms of our values and mission. Moreover, with the expansion of our group and the number of activities, we initiated committees focusing on membership, website, social media, symposiums and workshops, webinars, journal club, outreach, sponsorship, and translations. Despite having leaders for each committee, the main function of leadership is to facilitate communication between groups, and the overall structure is quite horizontal. Each year, we renew the leadership positions following nominations open to all active and passive members. We see this approach as making the temporary motivation permanent and honoring the members by acknowledging their efforts. Circulation in the community provides productivity and permanence. In addition to this, members can easily criticize the system, propose regulations, and even make permanent changes despite the existence of a formal chairship within RSG-Turkey. Meetings are open to all active members who can participate in decision-making. Decisions are not finalized without the consent of the majority. This allowed us to create a democratic and creative society with different views, in which everyone can express their opinions freely. This helped us increase the capacity for tolerance and respect in the RSG-Turkey. As a result, the internal conflicts in the RSG-Turkey were resolved efficiently and quickly. Hence, members show respect and are kind to each other not for their position/leadership but because they are colleagues and unite with a common goal.

The second critical point is the success of the task distribution within the group and ensuring its continuity through a good structure and effective communication. In RSG-Turkey, each active member shares the committees they want to join, and the leaders introduce the new members to the tasks and the overall flow of each committee. One of the primary responsibilities of the leaders is to communicate when the workload cannot be distributed reasonably between the active members, and there is a need for help. In RSG-Turkey, sustainability of the society and events in the long run is more valuable than the number of events, and thus, a reasonable workload is our primary goal. Keeping in mind that academic societies are mainly run by volunteers who already have an intense workload puts the wellbeing of the members first and benefits the society in the long run. It is quite normal that some tasks are not completed on time due to the busy schedules of the members. Thus, it is essential to plan the events considering some buffer time or to have enough members in each group so that they can transfer their duties to the others. If completion of a task is not possible by anyone in a given timeframe, one should remember it is a voluntary duty, and everyone might have other priorities in their life. Accepting these kinds of delays with understanding is the key in the community. After years of experience, we now established that long-lasting collaborations within our society are more valuable than arbitrary short-term goals.

## Lesson 3: Spend time on establishing effective communication, time, and project management structure

We have active members not only from different cities in Turkey but across the globe. Indeed, even the authors of this article are all from different universities and cities spanning five different countries and four time zones. We overcome this challenge by adopting online communication platforms. All events are organized through video calls and instant messaging platforms, allowing different channels for different purposes. We utilize online platforms for internal meetings, organizing webinars, workshops, and student symposiums, sharing and broadcasting our events using free online platforms, career talks with the academics, and most often, planning and tracking group activities.

The easy adoption of technology by everyone in the group ensures that the work is not interrupted and that the activities are carried out on time and with high participation. Cloud-based file sharing and storage methods facilitate keeping track of the current events and tasks, and more importantly, they help maintain a long-term memory within the society. The fact that there is a joint working folder where the chairs and active members can find the activities, meeting notes, symposium plans, and reports over the years enables the members to learn what has been achieved or not achieved in the past. These folders transfer the years-long experience to the new volunteers.

Advances in technology make digitalization a part of our lives. Digital transformation in communication has increased our productivity in using our time efficiently and being more systematic in our studies. We heavily use online platforms for project and time management through note-taking tools that allow real-time collaboration, task management tools, instant messaging services, and internal and shared calendars. We are available across multiple social media accounts to make sure potential members can reach us easily. We are also present on github ( github.com/rsgturkey) and share workshop materials openly. We believe adopting easy-to-use and free online platforms for communication and knowledge-sharing brings us one step closer to our dream of a genuinely accessible open scientific community.

## Lesson 4: Each challenge is an opportunity

**Figure 4.**
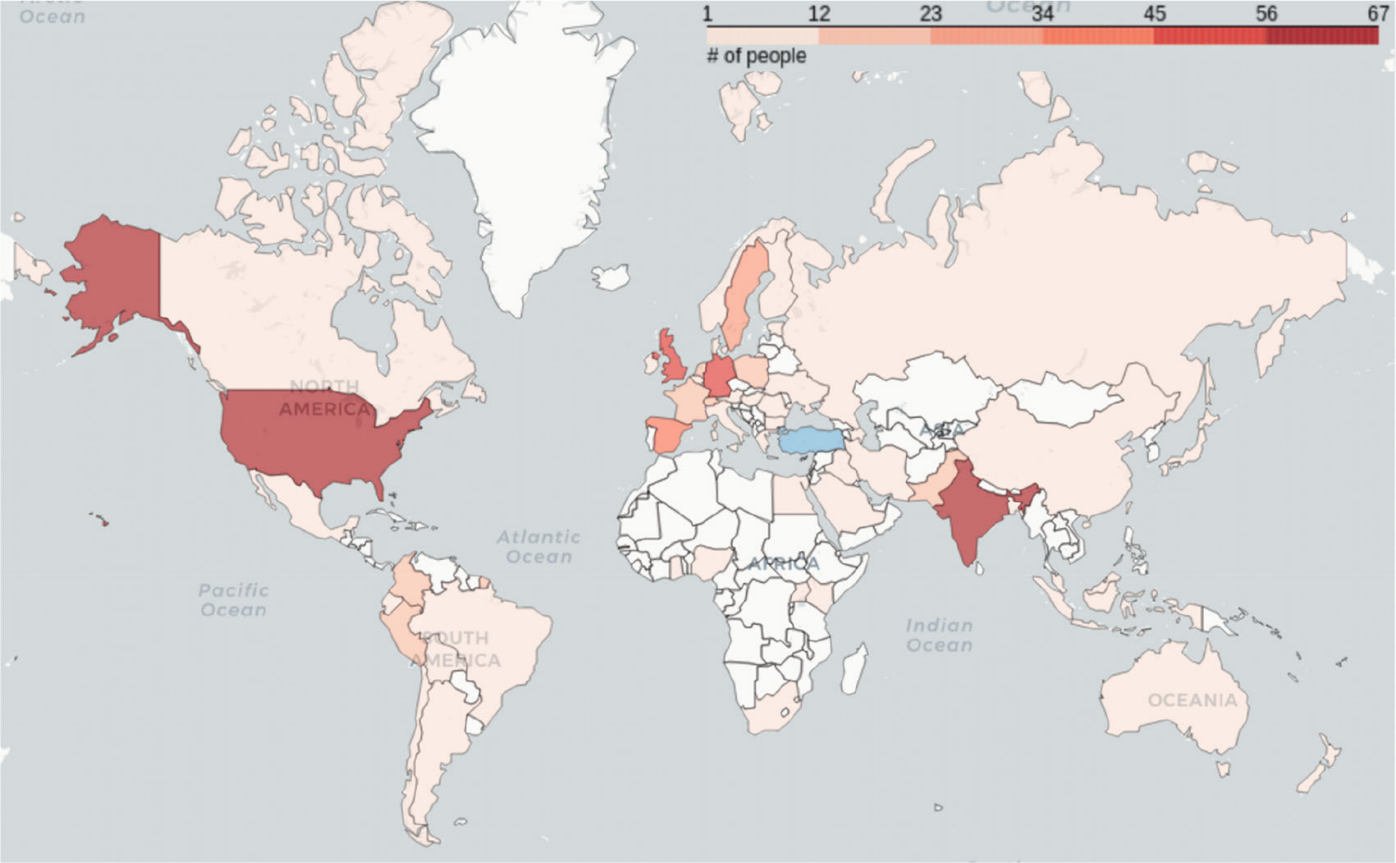
Distribution of people accessing RSG-Turkey webinar series worldwide. Turkey is excluded from the color scale and represented in blue.

Over the years, through many trials and errors, we realized that the main ingredient of the long-term sustainability of new initiatives and events is the need. We have acted on many ‘great’ ideas that were well planned and creative. However, those that did not timely address a need in the scientific society did not survive. Others, on the other hand, despite technical challenges and naivety in the planning steps, have been well received. For example, while the rest of the world cherishes exposure to top-notch science and scientific dialogue, students in countries with limited financial and human resources or a low number of international events have limited opportunities. Recognizing our need to be more exposed to science and have discussions around it, in 2013, we initiated the RSG-Turkey webinar series. At the time, even online learning platforms were just emerging, and releasing video recordings of the talks was not a common practice. Despite the connection issues and unfamiliarity of giving online talks, our events attracted many participants. The initiative contributed to the knowledge of students, helped them virtually interact with the speakers, and also facilitated a scientific culture with an emphasis on open science. In a short time, we realized our webinars were attended not only by students in Turkey but gained worldwide attention. To date, 63 countries have attended our webinars ( Figure 4). Now, we utilize this worldwide accession as an opportunity to share exciting science happening in Turkey to the globe and host many ECRs from Turkey as well. As this initiative spread, it started to benefit many people and inspire other communities. In this direction, we organized a common webinar series with RSG-Colombia and contributed to the ISCB-SC to launch its own webinar series. Furthermore, our experience has been fundamental to helping the entire ISCB-SC organization to transition to virtual meetings over the last two years during the Covid19 pandemic, which was essential to ensure the continuation of scientific events and growth of the ISCB-SC. ^8^^–^^10^ In short, what seemed like an adverse condition at the beginning led to an exciting journey and inspiration to others. Another example that stand-out is the difficult times we experienced during the Covid19 pandemic over the last two years (2020-2021). During this period, we organized more than 30 virtual meetings, including group meetings, networking meetings, and virtual events organizations. We hosted many researchers within and beyond Turkey and started new activities. We launched outreach activities and organized a few live broadcasts on Youtube to discuss the projects of high school students who have achieved national and international success in the field of computational biology. We are now inviting these high school students to become members of the RSG-Turkey. We also organized a Covid19 hackathon, providing a platform to collaborate and innovate to resolve pandemic-related data analysis challenges. Despite being the first attempt of the society to arrange a hackathon, the participants enjoyed the experience and continued their project as part of an international hackathon, ^11^ and published their findings as a preprint. ^12^ As most of our universities and institutes required us to strictly work from home, we required a new platform to discuss advances in science and communicate. Therefore, we established a Journal Club by making the best use of online platforms. In this way, we hope that we also provide an opportunity to improve the presentation and scientific communication skills of our members. Despite the tough conditions, we found support within our community, and we strive to adopt a proactive mindset and overcome future challenges ahead.

## Lesson 5: Collaborate!

Today, with the expansion of knowledge, collaboration in science and research is a must and the communication and collaboration skills of researchers become more and more important. Scientists come together to exchange ideas, knowledge, and expertise. These collaborative networks increase the impact and visibility of research. More importantly, joint projects result in new perspectives and lead to increased creativity and soundness. We believe our societal projects are not different. Being inspired by this, our group has thrived through both internal and external collaborations.

Since our early days, we have collaborated with the largest bioinformatics conference in Turkey, HIBIT, and organized our annual symposiums as a satellite meeting. This provided us with credibility and resources, while we contributed with a more student-focused program, including career talks and networking events. Similarly, we collaborated with multiple regional societies and associations (EkoEvo, MolBioGen, mbgtürkiye) and universities (Middle East Technical University, Kadir Has University, Gebze Technical University, Sabancı University, and Hacettepe University) to organize webinars and workshops. Through our positive dialogue with the local companies, we also managed to secure multiple sponsorships for our events, and we strive to make all our events free to attend by all students.

Despite being the regional student group, throughout our journey, we thrived by making the best use of being a part of a world-renowned international association. ISCB-SC has been supporting the RSGs both financially and through guidance. More importantly, despite being fully autonomous, RSGs are required to report on activities to the ISCB-SC, which provides additional credibility on the international ground. Moreover, we strive to collaborate with other RSGs, as exemplified by the joint webinars program with RSG-Colombia and planned activities with RSG-Pakistan and RSG-Denmark. Our international collaborations are not limited to the RSG network. We have cooperated with international societies such as Asia Pacific Bioinformatics Network, an ISCB affiliate, for the InSyB2021 bioinformatics symposium and Ostrava University for the Python programming workshop for bioinformatics held in Turkey.

Today, RSG-Turkey acts as a bridge between several universities and societies in computational biology. Thanks to all these collaborations and initiatives, we have increased our visibility and completed our academic structure with a reliable community vision that is open to opportunities and collaborations. All these bring along many benefits not only for RSG-Turkey, but also for the students who follow us closely, such as the opportunity to improve their social and technical skills, to find internships/jobs by networking, and to meet many scientists in the field of computational biology to have ideas and insights about their career. Moreover, these activities provided a training ground to our active members to improve their communication and collaboration skills, which are a crucial part of research life.

## Conclusion

One of the remarkable aspects of the 21 ^st^ century is the accessibility and affordability of data generation and analysis, which has led to the start of a new era focused on big data. In this environment, interdisciplinary work is not an advantage but a must for scientific progress. As the RSG-Turkey, we strive to support and contribute to the training of future generations of computational biologists and bioinformaticians to bridge the gap and satisfy the need for expert researchers in the field. Throughout our ten-year-long journey, we observed that our overall mission and values, community structure, tools for effective communication and project management, proactive mindset, and collaborations were key to our success. We believe that being part of an academic society and experiencing this in the early years facilitate the development of soft skills required in research, provide self-confidence and motivation, and contribute to collaborative research culture. Today, the first members of RSG-Turkey grew together to become senior postdocs and junior PIs that are all connected as part of a large, collaborative network. With the new generations of ECRs becoming independent researchers connected through the RSG-Turkey network in the future years, the benefits of creating RSG-Turkey will be larger than what we see today. We are eager to find out the long-term influence of this network on the academic society within and beyond Turkey. We hope that our experiences can provide inspiration and guidance to others towards establishing successful long-standing academic societies.
